# Secondary spontaneous pneumothorax and bullous lung disease in cannabis and tobacco smokers: A case-control study

**DOI:** 10.1371/journal.pone.0230419

**Published:** 2020-03-30

**Authors:** Alessandro Stefani, Beatrice Aramini, Carlo Baraldi, Lanfranco Pellesi, Giovanni Della Casa, Uliano Morandi, Simona Guerzoni

**Affiliations:** 1 Thoracic Surgery Unit, University of Modena and Reggio Emilia, Reggio Emilia, Italy; 2 Toxicology Unit and Drug Abuse Center, University of Modena and Reggio Emilia, Reggio Emilia, Italy; 3 Department of Radiology, University of Modena and Reggio Emilia, Reggio Emilia, Italy; University of Calfornia San Francisco, UNITED STATES

## Abstract

**Background:**

The notion that smoking cannabis may damage the respiratory tract has been introduced in recent years but there is still a paucity of studies on this subject. The aim of this study was to investigate the relationship between cannabis smoking, pneumothorax and bullous lung disease in a population of operated patients.

**Methods and findings:**

We performed a retrospective study on patients operated on for spontaneous pneumothorax. Patients were divided into three groups according to their smoking habit: cannabis smokers, only-tobacco smokers and nonsmokers. Cannabis lifetime exposure was expressed in dose-years (1d/y = 1 gram of cannabis/week for one year). Clinical, radiological and perioperative variables were collected. The variables were analyzed to find associations with smoking habit. The impact of the amount of cannabis consumption was also investigated by ROC curves analysis.

Of 112 patients, 39 smoked cannabis, 23 smoked only tobacco and 50 were nonsmokers. Median cannabis consumption was 28 dose/years, median tobacco consumption was 6 pack/years. Cannabis smokers presented with more severe chronic respiratory symptoms and bullous lung disease and with a higher incidence of tension pneumothorax than both tobacco smokers and nonsmokers. Cannabis smokers also developed a larger pneumothorax, experienced prolonged postoperative stay and demonstrated a higher incidence of pneumothorax recurrence after the operation than nonsmokers did. The risk of occurrence of chronic respiratory symptoms and bullous lung disease in cannabis smokers was dose-related.

**Conclusions:**

Cannabis smoking seems to increase the risk of suffering from respiratory complaints and can have detrimental effects on lung parenchyma, in a dose-dependent manner. Cannabis smoking also negatively affected the outcome of patients operated for spontaneous pneumothorax. A history of cannabis abuse should always be taken in patients with pneumothorax. There may be need for a specific treatment for pneumothorax in cannabis smokers.

## Introduction

Cannabis is the most commonly used illicit drug worldwide and the second most commonly smoked substance after tobacco. In Europe 17.5 million of young adults (15–34 years) used cannabis in 2019 (14.4%), while 91.2 million Europeans aged between 15 and 64 years (27.4%) reported having used cannabis at least once in their lifetime [1]. The consumption of cannabis has increased over the last decades, and this increase is partly due to the reduction in the perceived risks of smoking cannabis in adolescents and young adults [2]. Thus the impact of cannabis on the health of the general population is probably underestimated.

Many studies have been conducted on the mental health effects of cannabis smoking, but limited information is available on the respiratory and pulmonary effects. The notion that cannabis may damage the respiratory tract and the lung has been introduced in recent years [3,4]. Some respiratory consequences have been documented, such as chronic obstructive pulmonary disease symptoms [5,6] and bullous emphysema [7,8]. There are few studies on the association between cannabis smoking and spontaneous pneumothorax and a relationship is less clear, although a causative link has been suggested [7,9,10,11]. Most studies on this topic are case reports or small case-series, which mainly investigated the correlations between lung emphysema and pneumothorax. This lack of data may be because the analysis is based on a very specific population and because there are difficulties in collecting accurate information from individuals who can be unwilling to provide details about the use of an illicit substance. However, the relationship between cannabis smoking and the presentation of pneumothorax or the outcome after surgery has never been addressed.

The comparison of respiratory effects between cannabis and tobacco smoking is also a topic of interest. Some authors have suggested an additive effect of tobacco as a cause of lung damage or pneumothorax in cannabis smokers [10,12]. This suggestion is reasonable, given that tobacco smoking increases the risk of pneumothorax up to 20-fold, in a dose-dependent manner [13]. Again, very few studies have explored this association.

The aim of this retrospective study was to investigate the relationship between cannabis smoking and pneumothorax in a population of subjects who underwent surgical treatment for pneumothorax, focusing on clinical, radiological and perioperative features. These findings were compared with those of two other groups of patients operated for primary spontaneous pneumothorax (PSP) during the same period, non-smokers and only-tobacco smokers. The possible interactions between cannabis and tobacco smoking were also analyzed.

## Materials and methods

The study was approved by the local Ethics committee. The approval number is CE 1098-170/13. Written informed consent was obtained from all patients.

All patients who underwent an operation for PSP at our Institution between January 2009 and December 2018 were retrospectively reviewed from a prospectively maintained database. Surgery was indicated for recurrent pneumothorax or in cases of prolonged air leak lasting more than 5–6 days after intercostal drainage.

Only patients who had a pre-operative CT-scan of the chest were included in the study. We usually perform a high-resolution CT-scan after the first episode of pneumothorax, or before operating on a patient with prolonged air leak, to study the lung parenchyma for emphysematous changes, blebs or bullae. Therefore, the majority of our operated patients had a CT-scan although, in light of the aforementioned indications, in our series of patients CT-scans were performed in different times compared to the time of the operation. Patients presenting with secondary pneumothorax (e.g. lymphangioleiomyomatosis or catamenial pneumothorax) or older than 40 years were excluded, to reduce bias from unacknowledged underlying diseases and because previous studies showed that PSP rarely occurs in patients older than 40 years (10). Of course, the presence of bullae or others emphysematous changes at CT-scan were not considered among the diseases determining the exclusion from the study.

### Clinical and toxicological features

The clinical variables we recorded were sex, age at operation, chronic respiratory symptoms (cough, dyspnea, phlegm, wheeze, pharyngitis or asthma) and symptoms at the onset of pneumothorax (chest pain and/or dyspnea). In patients with multiple ipsilateral episodes before the operation, the symptoms related to the most severe event were considered. Lung function tests were not usually performed before operation, therefore this information was not available.

Because for most patients toxicological information retrieved from the clinical charts were not adequate for the purposes of the study, all patients were contacted, by telephone interviews or ambulatory visits, and were requested to fully disclose their inhalation history. Smoking history was expressed through the measure of pack-years (p/y, one pack-year = 20 tobacco cigarettes per day for 1 year). Regarding cannabis consumption, the patients were asked to disclose how many grams of cannabis per week he/she smoked and for how long, as well as the type and method of cannabis use. Lifetime cannabis consumption was expressed in dose-years (d/y): one dose-year was defined as smoking 1 gram of cannabis per week for one year. No distinction was made between marijuana and hashish. Subjects who smoked cannabis in a form other than a joint (e.g. pipes or bongs) were excluded, as well as those who disclosed abuse of inhaled substances other than cannabis or tobacco. Urine toxicological examinations were not routinely performed on admission.

### Radiological features

Chest roentgenograms and CT-scans of the chest of all patients were retrospectively reviewed. All images were available on screen as digital images on the hospital picture-archiving communication system (PACS). The size of pneumothorax was estimated on upright posteroanterior chest radiograph, at full inspiration. Pneumothorax was defined as complete when the visceral and parietal pleurae were completely separated from the apex to the base of the hemithorax, while it was defined as partial in all the other cases. In patients with multiple ipsilateral episodes of pneumothorax before the operation, the radiograph showing the largest pneumothorax was considered. A tension pneumothorax was defined when a contralateral mediastinal shift was detected.

**Fig 1:**
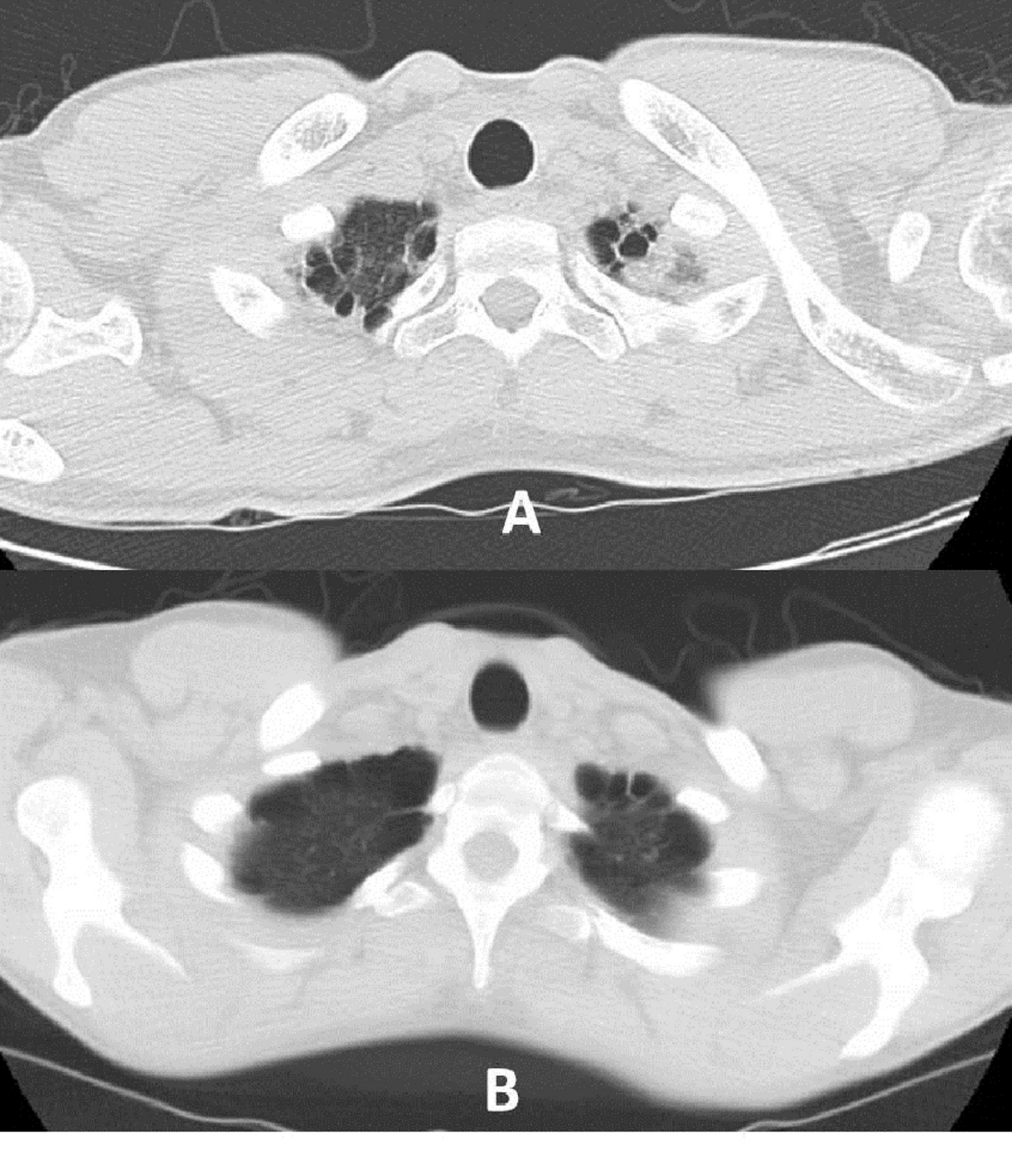
CT scan showing high-grade DSS: A) grade 5 DSS (multiple bilateral blebs), and B) grade 6 DSS (multiple bilateral bullae).

CT-scans were reviewed for the presence of air-containing pulmonary lesions. The lesions were considered as blebs if they had a diameter of ≤1cm, bullae if the diameter exceeded 1cm. We applied a scoring system to assess the severity of the lesions (dystrophic severity score–DSS), as already described by other Authors and us [14,15]. In brief, different values were assigned according to the type (1 or 2 points for blebs or bullae, respectively), number (1 or 2 points for single or multiple lesions, respectively) and distribution (1 or 2 points for unilateral or bilateral lesions, respectively) of air-containing lesions. The final score ranged from 0 (no lesions) to 6 (multiple bilateral bullae). For the statistical analysis, the DSS was grouped into low-grade (DSSs from 0 to 4) and high-grade (DSSs of 5 and 6) (Fig 1A and 1B).

### Perioperative findings

All surgical procedures were performed through a video-assisted thoracoscopic approach (VATS), for bullectomy and/or resection of the lung apex in addition to pleural abrasion. The following variables were recorded: surgical indication (recurrence or prolonged air leak), operative time, number of stapler cartridges used for lung resection, conversion to thoracotomy, duration of chest drainage, length of hospital stay and postoperative complications. Prolonged air leak was considered when it lasted more than 5 days. A lung collapse larger than 2cm at the apex observed in the chest X-ray before discharge was considered an incomplete lung re-expansion. Data regarding perioperative blood loss were not available.

### Follow-up

Ipsilateral recurrence after operation was recorded. Cannabis and tobacco consumption after the operation were not further investigated. Follow-up was completed on July 2019.

### Statistical analysis

Descriptive analysis was expressed in terms of the frequency, median, mean and standard deviation (SD). Frequencies were compared using the χ^2^-test or Fischer’s exact test for categorical variables. Continuous variables were compared using the Student’s test or the analysis of variance. Ordinal variables were compared using nonparametric tests (Kruskal-Wallis). We divided the patients into 3 groups, regardless of the amount of lifetime consumption: cannabis smokers, with or without associated tobacco consumption (CaS), tobacco-only smokers (TS) and nonsmokers (NS). The groups were compared in a univariable and multivariable analysis. Continuous variables were dichotomized when needed and the median value of the whole population was used as the cut-off value. Multivariable analysis was performed using logistic regression; odds ratios (ORs) were calculated with 95% confidence intervals (CI). For multivariable analysis, because our study was focused on the effects of cannabis versus non-smoking or only-tobacco smoking, TS and NS were grouped and compared with CaS. The effect of the amount of cannabis exposure on the 39 CaS was explored for each variable using the Student’s test and, in cases of a significant difference was found, receiver-operating characteristic (ROC) curves were created. A value of p<0.05 was considered significant.

All analyses were performed using SPSS 25.0 statistical software (SPSS Inc, Chicago, IL).

## Results

One-hundred and forty-five patients were consecutively operated between 2009 and 2018. Of these, 112 (77%) had preoperative CT-scan of the chest and were included in the study. The characteristics of the whole population are reported in Table 1. Most patients experienced chest pain at the onset of pneumothorax (102/112, 91%), 58 patients (52%) experienced dyspnea and 49 (44%) experienced both symptoms. Twenty-nine patients had a DSS of 0 (26%), 21 patients of 3 (19%), 15 patients of 4 (13%), 28 patients had 5 (25%) and 19 patients had 6 (17%). Thirty-nine patients were cannabis smokers (35%), 23 were only-tobacco smokers (20%) and 50 were nonsmokers (45%). Thirty-seven out of 39 cannabis smokers (95%) were also tobacco smokers. Median lifetime cannabis consumption was 28 d/y (mean 48, SD 57, range 0.5–250 d/y), while median tobacco consumption was 6 p/y (mean 7.5, SD 6.1, range 0.2–20) in the TS group and 6 p/y (mean 6.9, SD 5.1, range 0–20) in the CaS group. There was no difference in tobacco exposure between CaS and TS (p = 0.691). There was a significant difference in the distribution of smoking habit between sexes (p = 0.002): a total of 39% of men (35/89) smoked cannabis versus only 17% of women (4/23), conversely 48% of women (11/23) smoked tobacco versus only 13.5% of men (12/89).

**Table 1:** Characteristics of the whole population and univariable analysis. The overall comparison among the three groups is shown.

Variable	N (%)				P
	Overall (n = 112)	CaS (n = 39)	TS (n = 23)	NS (n = 50)	
Sex					
male	89 (79.5)	35 (90)	12 (52)	42 (84)	0.002
female	23 (20.5)	4 (10)	11 (48)	8 (16)	
Age (years)					
Mean (SD)	25 (7.1)	26.4 (6.6)	27.1 (7.6)	22.7 (4.5)	0.012
Chronic respiratory symptoms					
yes	28 (25)	22 (56)	4 (17)	2 (4)	<0.001
no	84 (75)	17 (44)	19 (83)	48 (96)	
Symptoms at pneumothorax					
pain or dyspnea	111 (99)	21 (55)	11 (48)	30 (60)	0.620
both	49 (44)	17 (45)	12 (52)	20 (40)	
Pneumothorax size					
partial	43 (38)	9 (23)	7 (30)	27 (54)	0.008
complete	69 (62)	30 (77)	16 (70)	23 (46)	
Tension pneumothorax					
yes	25 (22)	15 (39)	6 (26)	4 (8)	0.003
no	87 (78)	24 (61)	17 (74)	46 (92)	
CT-scan DSS					
low-grade (0–4)	65 (58)	4 (10)	15 (65)	46 (92)	<0.001
high-grade (5–6)	47 (42)	35 (90)	8 (35)	4 (8)	
Indications for surgery					
recurrence	96 (86)	32 (82)	18 (78)	46 (92)	0.214
prolonged air leak	16 (14)	7 (18)	5 (12)	4 (8)	
Operative time (min)					
Mean (SD)	67 (19)	70 (20)	70 (26)	63 (15)	0.212
N° of stapler cartridges					
Mean (range)	2.7 (1–6)	3.1 (2–5)	2.9 (1–6)	2.3 (1–5)	0.001
Postoperative complications					
yes	26 (23)	11 (28)	6 (26)	9 (18)	0.493
no	86 (77)	28 (72)	17 (74)	41 (82)	
Duration of drainage (days)					
Mean (range)	2.9 (1–19)	3.6 (1–19)	2.9 (2–8)	2.6 (2–10)	0.135
Length of p.o. stay (days)					
Mean (range)	4.0 (2–20)	4.7 (2–20)	4.0 (3–8)	3.5 (2–11)	0.076
Postoperative recurrence					
yes	11 (10)	7 (18)	2 (9)	2 (4)	0.088
no	101 (90)	32 (82)	21 (91)	48 (96)	

Table 1 shows the results of the univariable analysis comparing the three groups.

An additional analysis was performed by matching one group to each other, to investigate the specific associations within each variable (Table 2). This analysis showed that CaS presented a higher incidence of chronic respiratory symptoms, bullous lesions and tension pneumothorax compared to both TS and NS. CaS also showed a larger pneumothorax, prolonged postoperative stay and higher incidence of recurrence compared with NS but not compared with TS. TS showed a higher incidence of tension pneumothorax and bullous lesions compared to NS. More stapler cartridges were used for bullectomy in the CaS and TS groups with respect to NS group.

**Table 2:** Univariable analysis: Matched-paired comparison between groups. Data are expressed as ORs, 95%CI and Pearson’s index. Continuous variables have been dichotomized. Only comparisons with statistical significance are reported.

VARIABLE	CaS vs.TS (n = 62)	CaS vs.NS (n = 89)	TS vs.NS (n = 73)
Chronic respiratory symptoms (yes vs no)	6.1(1.7–21.4) p = 0.002	31.0(6.5–146.7) p<0.001	5.0(0.8–29.9) P = 0.053
Pneumothorax size (complete vs partial)	1.4(0.4–4.7) 0.528	3.9(1.4–10.4) 0.003	2.6(0.9–7.9) 0.063
Tension pneumothorax (yes vs no)	3.07(0.96–8.60) 0.047	7.19(1.94–26.57) <0.001	4.06(1.06–17.05) 0.038
CT-scan DSS (high-grade vs low-grade)	16.41(3.13–86.06) <0.001	100.63(7.95-∞) <0.001	6.13(1.47–25.53) 0.004
Stapler cartridges (n) (>2 vs ≤2)	1.18(0.35–3.92) 0.790	3.61(1.36–9.59) 0.005	3.07(1.01–9.45) 0.039
Length of p.o.stay (days) (>3 vs ≤3)	1.87(0.75–3.57) 0.192	2.97(0.98–6.43) 0.048	1.26(0.45–2.40) 0.393
Postoperative recurrence (yes vs no)	2.30(0.42–12.48) 0.322	5.25(1.09–28.4) 0.031	2.29(0.29–17.74) 0.416

Focusing the analysis on CT-detected lesions, we found that almost all patients with a DSS = 6 were CaS (18/19, 95%); accordingly, 18/39 CaS presented with a DSS = 6 (46%) while only 2 CaS had a DSS = 0 (5%). Conversely, no NS had a DSS = 6, while 21/50 NS had a DSS = 0 (42%).

Seven patients experienced postoperative prolonged air-leak, of whom 5 were CaS. An incomplete lung re-expansion was detected in 12 patients: 4 were CaS, 2 were TS and 6 were NS. No differences were found among the groups regarding the type of complications. Conversion to thoracotomy was performed in 5 patients (4.4%), of whom 3 were CaS, one was a TS and one was a NS.

The results of the multivariable analysis are shown in Table 3. Cannabis smokers presented a higher risk of suffering from chronic respiratory symptoms (OR 6.20), showing high-grade DSS (OR 41.03) and developing tension pneumothorax (OR 10.7) than the other patients (TS and NS grouped together).

**Table 3:** Multivariable analysis: CaS were matched against TS and NS grouped together. Continuous variables have been dichotomized.

Covariates	CaS vs TS + NS		
	OR	95% CI	P
Sex (males vs. females)	2.13	0.19–24.48	0.544
Age (>24 vs ≤24 years)	1.24	0.28–5.02	0.773
Chronic respiratory symptoms (yes vs. no)	6.2	1.23–31.29	0.027
Symptoms at pneumothorax (yes vs. no)	0.26	0.04–1.77	0.171
Pneumothorax extension (complete vs. partial)	3.81	0.61–10.72	0.451
Tension pneumothorax (yes vs. no)	10.7	1.17–98.11	0.036
DSS (high-grade vs. low-grade)	41.03	7.04–239.25	<0.001
Operative time (>60 vs. ≤60 min.)	1.44	0.26-.3.17	0.419
Number of stapler cartridges (≤2 vs. >2)	1.08	0.22–5.35	0.928
Postoperative complications (yes vs. no)	2.7	0.45–16.41	0.371
Duration of drainage (>2 vs. ≤2 days)	1.17	0.18–7.37	0.907
Length of p.o. stay (>3 vs ≤3)	2.39	0.42–9.35	0.386
Postoperative recurrence (yes vs. no)	5.72	0.59–57.52	0.163

OR: odds ratio; CI: confidence intervals.

The impact of the amount of cannabis consumption on clinical, radiological and perioperative features is shown in Table 4. The comparison of means indicated a significant correlation between cannabis exposure and both chronic respiratory symptoms and DSS. No other correlations were found.

**Table 4:** Distribution of cannabis consumption for each variable. The analysis was performed on the 39 cannabis-smokers. Cannabis exposure is expressed as a mean of dose/year.

Variables	Patients N (%)	Cannabis exposure (d/y)	P
Chronic respiratory symptoms			
no	17 (43)	17.8	0.001
yes	22 (57)	72.3	
Symptoms at pneumothorax			
pain or dyspnea	21 (54)	44.6	0.518
both	18 (46)	56.0	
Pneumothorax size			
partial	9 (23)	42.4	0.722
complete	30 (77)	50.5	
Tension pneumothorax			
no	24 (61)	48.9	0.999
yes	15 (39)	48.1	
CT-scan DSS			
low-grade (0–4)	4 (10)	22.3	0.045
high-grade (5–6)	35 (90)	51.1	
Operative time (min)			
≤60	18 (46)	49.6	0.890
>60	21 (54)	47.0	
N. stapler cartridges			
≤2	9 (23)	27.8	0.226
>2	30 (77)	54.3	
Postoperative complications			
no	28 (72)	48.3	0.976
yes	11 (28)	47.7	
Duration of drainage (days)			
≤2	20 (51)	35.8	0.176
>2	19 (49)	61.2	
Length of p.o. stay (days)			
≤3	21 (54)	46.9	0.883
>3	18 (46)	49.6	
Postoperative recurrence			
no	32	43.0	0.757
yes	7	48.5	

**Fig 2:**
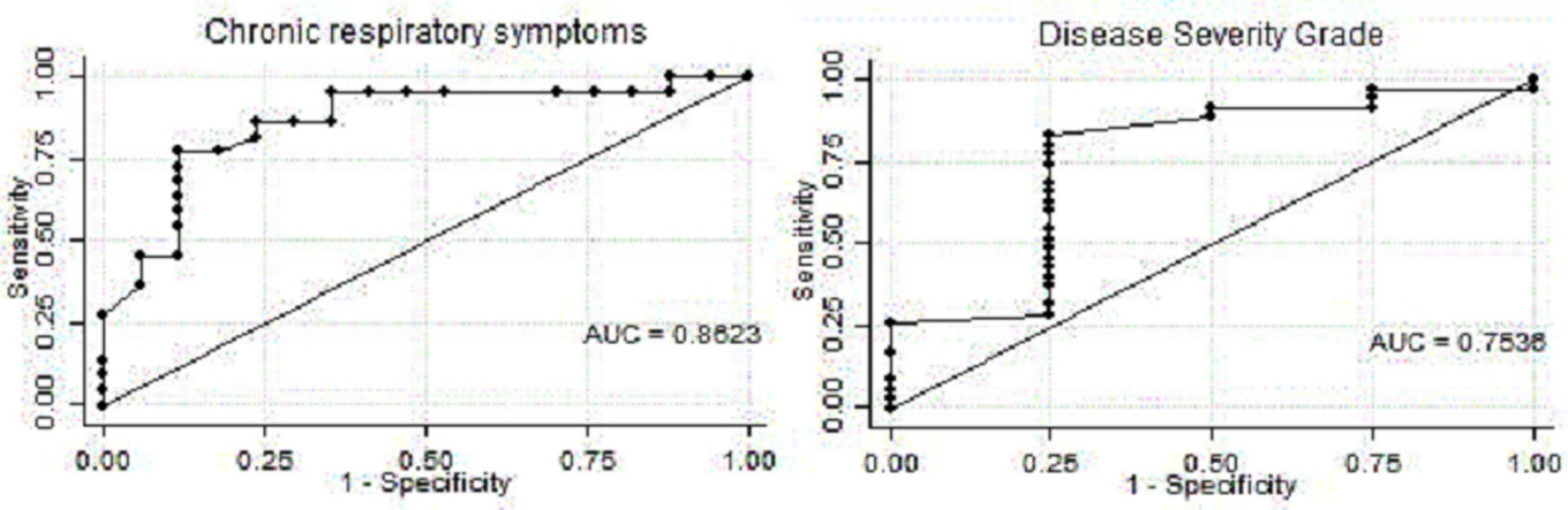
ROC curves for chronic respiratory symptoms and DSS.

ROC curves are reported in Fig 2 and show an 86% probability of suffering from chronic respiratory symptoms at 29 d/y and a 75% probability of having high DSS at 6.2 d/y (Table 5).

**Table 5:** Cut-point of cannabis consumption predicting the outcome. Area under-the-curve, sensitivity and specificity at the cut-point are reported.

VARIABLE	Cannabis consumption cut point (d/y)	AUC	Sensitivity	Specificity
Presence of chronic respiratory symptoms	29.01	0.862	88%	83%
High-grade DSS	6.25	0.753	83%	75%

## Discussion

It is well known that tobacco smoking leads to an increased risk of developing pneumothorax [13], but limited information is available on the relationship between cannabis and pneumothorax. In a study on a large sample of young Danish people, Olesen found that subjects who smoked both cannabis and tobacco had a significantly higher risk of having a spontaneous pneumothorax compared to both NS and TS [10]; however, the study was not designed to investigate the clinical features of pneumothorax. The few other studies on this topic focused mainly on emphysematous lesions and the histopathological features of cannabis smokers who developed pneumothorax [17]. To the best of our knowledge, our study is the first to investigate the clinical, radiological and perioperative aspects of pneumothorax in a population of cannabis smokers.

The high prevalence of cannabis smokers in our population (35%) is not surprising. According to a recent Report of the European Monitoring Centre for Drugs [1], 14% of young adults (15–34 years) used cannabis in the 2016. The prevalence of cannabis smokers seems to be even higher in patients developing a pneumothorax. In fact, in the study of Olesen, 34.9% of subjects in the pneumothorax group smoked cannabis within the last year versus 11.5% in the whole population [10]. Moreover, in accordance with common observations [1,10], also in our series we found that CaS were young and were mainly males.

We observed that CaS experienced a higher incidence of respiratory symptoms than both NS and TS, in univariable and multivariable analysis. The relationship between cannabis smoking and respiratory symptoms was the first respiratory effect of cannabis that was investigated and demonstrated [4,5,12,16,17]. In a study on 7000 adults, Moore and colleagues showed that the use of marijuana was accompanied by respiratory symptoms and that subjects who smoked both marijuana and tobacco had a greater prevalence of symptoms than those who smoked only tobacco [5].

The correlation among cannabis smoking and pneumothorax is still debated. In 2006 a review by Tan and coll., based on 10 patients in four reports, stated that the existing data were unable to confirm a causative link [18]. Since then other case reports, which have been collected in a review by Underner and coll. [17], and three case series have been published. Beshay and coll. [7], Fiorelli and coll. [9] and Ruppert and coll. [19] reported 102, 153 and 83 patients respectively who presented with spontaneous pneumothorax, of whom 17 (17%), 13 (8%) and 32 (38%) respectively smoked cannabis. In these studies CaS showed an higher incidence of lung bullae detected by CT-scan with respect to cannabis nonsmokers (tobacco smokers or nonsmokers). Bullous emphysema due to cannabis was mainly paraseptal, and hence distinct from the more uniform distribution of centrilobular emphysema, which is typically associated with tobacco smoking. In both cannabis and tobacco smokers emphysema was mainly located in the upper lung fields. According to these Authors, we also found a relationship between cannabis smoking and lung bullae, which were located in the upper lobes in all our patients. The use of a semiquantitative method to measure the degree of bullous lesions in the CT-scan is an original approach, and it led us to find a striking difference between CaS and NS, but also a difference between CaS and TS.

However, none of the aforementioned studies investigated the aspects of pneumothorax. In our study, a larger pneumothorax and an impairment in postoperative outcomes were observed in CaS with respect to NS. The more severe lung disease observed in CaS could be responsible for the larger pneumothorax. In fact, we found a significant correlation between DSS and pneumothorax size: 34/65 patients (52%) with DSS of 0–4 experienced a complete pneumothorax versus 35/47 patients (75%) with DSS of 5–6 (p = 0.017, OR 2.66). This relationship was also observed by Kawaguchi and coll. [20]. In our series, the size of pneumothorax did not seem to affect the occurrence of symptoms, which was comparable among groups. The presence of bullous lesions may also partly explain the larger number of stapler cartridges used for bullectomy in CaS: larger bullae needed wider excisions, as also observed by Beshay [7]. Nevertheless, neither the lung bullae nor the number of cartridges prolonged the operative time, which was similar in both groups. CaS also experienced prolonged postoperative stay compared with NS. The overall incidence of complications was comparable, but prolonged air leak occurred more frequently in CaS than in NS (5 patients versus one, respectively). This result, in addition with an average drainage time one day longer for CaS (p = 0.077), may partly explain the difference in hospital stay between the two groups. Stapler application on a dystrophic lung parenchyma may be partially responsible for the prolonged air leak in CaS. Conversion to thoracotomy seemed to occur more frequently in CaS than in NS (3 patients versus one, 7.8% versus 2% respectively), but the number of events was too small to support a reliable analysis. Most importantly, we found a higher incidence of postoperative recurrence of pneumothorax in CaS than in NS. Asano observed a relationship between the detection of blebs/bullae on CT-scans and a low recurrence rate, perhaps because such identification led to easier intraoperative detection and appropriate resection [21]. Conversely, in our series, the presence of major bullous changes and the difficulty of properly resecting all lesions may be hypothesized as causes of recurrence in CaS. Nonetheless, we did not find correlations between any other variable and recurrence but this result could be due to the limited number of events (only 11 cases of recurrence).

Because the majority of people who smoke cannabis also smoke tobacco, a common issue when investigating pulmonary hazards of smoking habit is discriminating the effects of cannabis from those of tobacco [7,10,16]. This introduces a confounding factor in any study and the issue cannot be overcome by a controlled randomized trial comparing only-tobacco smokers to only-cannabis smokers. However, some studies on the characteristics of cannabis smoking has led to the perception that cannabis use could result in more severe pulmonary lesions compared to tobacco smoking [16]. In fact, although smoke from the two substances is virtually identical (other than psychoactive compounds), a draw on a cannabis joint involves a two-thirds larger puff volume, a one-third greater depth of inspiration and a fourfold longer breath-holding time than a draw on a tobacco cigarette. Moreover, the lack of filter tips on cannabis joints leads to an average fourfold greater delivery of tar [16]. Hence, it is reasonable that the barotrauma, combined with the direct toxicity of the inhaled particulate, may be responsible for major lung damages and pneumothorax.

In our population the majority of CaS also smoked tobacco (37/39, 95%). Of note, CaS used similar amount of tobacco as tobacco-only smokers (median 6 pack/years in both groups). Therefore, we can argue that smoking cannabis induces a severe detrimental effect, regardless of the concomitant use of tobacco, for those variables in which we found a significant difference, not only between CaS and NS, but also between CaS and TS, that is chronic respiratory symptoms, bullous lung disease and tension pneumothorax (Table 2). The results of multivariable analysis, in which TS and NS were grouped together, may confirm this hypothesis. Conversely, when investigating length of stay and recurrence, we did not find a significant difference between CaS and TS, although CaS showed a two-fold higher incidence of recurrence and a 0.7 day longer postoperative stay with respect to TS (OR 2.30 and 1.87 respectively). No differences were observed, for these two variables, also between TS and NS and in the multivariable analysis, where CaS were compared to TS+NS. It is likely that the small sample sizes contributed to the lack of statistical significance, especially for variables in which absolute differences among groups were quite small. Anyway, the lack of significant difference between CaS and TS makes it difficult to discriminate whether the differences between CaS and NS in the length of stay and recurrence can be ascribed to the effect of cannabis *per se* or to an additive effect to tobacco, as previously suggested [12,17].

In this study we introduced an original method to measure lifetime cannabis exposure. Various methods have been proposed and the most commonly used is “joint/years”: joints/day times years of abuse [3,22]. However, because cannabis is purchased by weight (grams) and the number of joints obtained per gram is quite different among consumers, we have considered more adequate to express the consumption in grams. Furthermore, because cannabis consumption is frequently not regular during the week (most individuals smoke mainly or exclusively during the week-end), we considered more appropriate to adopt the week as unit of time. Therefore, we have proposed “dose/years” as the standard unit to assess lifetime exposure and, using this method, we investigated the impact of the amount of cannabis consumption. We found a dose-dependent effect of cannabis smoking with respect to respiratory symptoms (86% probability of suffering from respiratory symptoms for an exposure at 29 dose-years) and bullous lung disease (75% probability of having high-grade lesions at 6.25 dose-years). From our findings it seems that a relatively low consumption of cannabis may be sufficient to cause lung bullae, while chronic complaints could occur after a higher exposure. No significant dose-dependent effect was found for any perioperative feature, but this might be due to the small sample size of 39 patients. To date, few studies have investigated the dose-response relationships of cannabis smoking and none of these using statistical models, as in our study (Student’s test and ROC curves). Aldington reported a dose-response relationship of cannabis smoking with airflow obstruction, hyperinflation and respiratory symptoms [3], Beshay suggested that the period of smoking represents an important factor in the development of lung bullae [7].

We classified a priori all our patients as having a PSP. However, as cannabis smoking seems to present a strong association with lung bullous disease and spontaneous pneumothorax, one might argue that pneumothorax in cannabis smokers should be considered a *secondary* condition, as previously suggested [7,10]. This change of status is far from being a theoretical exercise and could lead to the proposal of a specific management for these patients, different from the standard treatment of PSP. Surgery after the first episode, the use of a more effective method of pleurodesis and the use of staple-line reinforcement may be considered as treatment options for pneumothorax in cannabis smokers.

This retrospective study has some limitations. It included only patients who underwent an operation for pneumothorax, thus symptoms and lung lesions may be more severe than in the general population. Therefore, our findings cannot be extended to the whole population of otherwise healthy cannabis smokers. The score for measuring bullous lesions in the CT-scan has been previously reported but it has not yet been validated. Also our method to measure lifetime cannabis exposure has not yet been validated. Because of the concomitant use of cannabis and tobacco, we could not definitively detect an isolated effect of cannabis, although the cross comparisons among the three groups suggested a predominant effect of cannabis for some variables. Small sample sizes may impair the reliability of the statistics, especially when the group of TS was included in the analyses. Finally, cannabis consumption was self-reported and could have been underestimated, because some individuals may have been reluctant to give details about smoking an illegal substance.

## Conclusions

Our study confirmed that cannabis smoking increases the risk of suffering from respiratory complaints and can have detrimental effects on lung parenchyma, in a dose-dependent manner. Cannabis smokers had a higher risk of developing a large pneumothorax and experiencing prolonged postoperative stay and recurrence compared to NS. Our results suggest a quite predominant effect of cannabis over tobacco in determining the different outcomes. Young people should be fully informed about the risks of cannabis smoking, and the public awareness of the health hazards of cannabis should be increased. A history of cannabis abuse should always be taken in patients with pneumothorax, prior to any intervention. If present, CT images should be obtained, to detect potential bullous lung disease. Some aspects of the treatment of spontaneous pneumothorax in cannabis smokers may be different than in patients with PSP and this could be the subject for future researches. Further studies on large samples of only-cannabis and only-tobacco smokers should be conducted, to finally clarify the respective role of cannabis and tobacco in determining respiratory hazards and pneumothorax.
